# Statistical Analysis of the Performance of MDL Enumeration for Multiple-Missed Detection in Array Processing

**DOI:** 10.3390/s150820250

**Published:** 2015-08-18

**Authors:** Fei Du, Yibo Li, Shijiu Jin

**Affiliations:** State Key Laboratory of Precision Measurement Technology and Instrument, Tianjin University, Tianjin 300072, China; E-Mails: dukemyy@tju.edu.cn (F.D.); shjjin@tju.edu.cn (S.J.)

**Keywords:** performance analysis, minimum description length (MDL), array processing, multiple-missed detection, source enumeration

## Abstract

An accurate performance analysis on the MDL criterion for source enumeration in array processing is presented in this paper. The enumeration results of MDL can be predicted precisely by the proposed procedure via the statistical analysis of the sample eigenvalues, whose distributive properties are investigated with the consideration of their interactions. A novel approach is also developed for the performance evaluation when the source number is underestimated by a number greater than one, which is denoted as “multiple-missed detection”, and the probability of a specific underestimated source number can be estimated by ratio distribution analysis. Simulation results are included to demonstrate the superiority of the presented method over available results and confirm the ability of the proposed approach to perform multiple-missed detection analysis.

## 1. Introduction

Source enumeration is a critical step in array signal processing and widely used in many scenarios [1]. The accuracy and the tendency of enumeration will significantly affect the performance of succeeding algorithms, such as direction-of-arrival (DOA) estimation [2] or blind source separation [3]. Minimum description length (MDL) criterion derived by Rissanen [4], or its equivalent criterion derived by Schwarz under the name of Bayesian information criterion (BIC) [5], is one of the most commonly used enumeration methods for its low complexity and asymptotic consistency which ensures a correct estimation as the sample size tends to infinity [6]. A lot of algorithms have been proposed to improve the MDL criterion for a performance promotion, low computation complexity or robustness in various environments [7,8,9,10,11,12,13,14,15]. Dayan and Rausley have presented a norm-based improved MDL (iMDL) algorithm in [7] by nonlinear rescaling of the sample eigenvalues and the corresponding normalized indexes. By using the training sequence of the desired signal, a minimum mean square error (MMSE) based MDL method has been developed by Huang in [8] to get a more accurate estimation of the source number. Huang and So have also employed the linear shrinkage estimation of noise subspace covariance matrix instead of sample covariance matrix in MDL criterion in [9] to achieve a more reliable detection in severe environments where the number of snapshots is comparable or even smaller than the number of sensors. For the cases at a small sample size, the probability density function of the sample eigenvalues has been taken into consideration in MDL as an essential supplement in [10,11]. To handle the coherent signals contaminated by colored noise, Zhen and Si [12] have whitened the sample eigenvalues to eliminate the inequality of eigenvalues caused by colored noise. Fishler and Poor [13] have proposed a robust-MDL (RMDL) method with proven consistency for source enumeration under non-uniform noise situations while Huang *et al.* [14,15] have improved MDL by introducing a multi-stage Wiener filter by using the filtered component variances or MMSE rather than the sample eigenvalues, which can offer computational simplicity and robustness to non-uniform noise.

Most of the above methods will give an accurate estimation of source number under the assumption of an infinite sample size. However, only a limited number of observations is available in practice particularly in the applications with latency requirements. Thus, the performance analysis of MDL at a finite sample size is of great practical value.

The statistical performance of MDL criterion for source enumeration has been extensively analyzed in [16,17,18,19,20,21,22,23,24,25,26,27]. Since the method is eigendecomposition-based, the statistical property of the sample eigenvalues has been investigated considerably. The distributions of the sample eigenvalues which were derived from the multivariate statistical theory [28], have been used by the authors in [16,17,18]. However, the performance estimations are found biased when the sample size is not sufficiently large. Recently, the random matrix theory [29] approach has been proposed to solve the enumeration problem in array processing in [30,31,32,33]. Asymptotic distributions of the sample eigenvalues have been given to rectify the bias by taking in the influence on signal eigenvalues from the noise subspace under the assumption of large dimension. For non-circular or non-Gaussian cases, statistical analysis has been made in [22,23] by taking the fourth-order statistics of the signals into consideration. In [34], the interactions between signal eigenvalues are considered by Lawley on the distribution analysis of the sample eigenvalues. The authors in [24,25,26,27] are able to predict the probability of underestimation in close accordance at a moderate sample size by a combination of different theories.

Although many analyses on the performance of MDL are available, few discuss the cases that the source number is underestimated by a number greater than one, which is denoted as “multiple-missed detection” here. Since the enumeration performance of MDL is signal-to-noise ratio (SNR) dependent, the source numbers estimated under varying noise levels may be different. This inconsistency in enumeration can be attributed to either the variation of actual source number or the disturbance of noise. By the statistical analysis on multiple-missed detection, the probability of a specific enumeration result can be estimated as an important reference for the attribution of the enumeration discrepancy.

In this paper, we propose a new procedure for the performance evaluation of MDL, which can predict the estimation results of MDL precisely at a finite sample size by considering the interactions between signal eigenvalues. A novel approach is also developed for the multiple-missed detection analysis. Thus the deterioration of enumeration performance with the degradation of SNR can be estimated.

The remainder of this paper is organized as follows: the problem formulations are given in Section 2, including the array signal model and the theoretical analysis of underestimation. Section 3 introduces the statistical analysis of underestimation by discussing the distributive property of the sample eigenvalues. Simulation results that illustrate the superior performance of the proposed method and the performance for multiple-missed detection are presented in Section 4. Finally, conclusions are drawn in Section 5.

## 2. Problem Formulation

### 2.1. Array Signal Model

Consider *q* narrowband far-field and incoherent sources impinging on a sensor array of *p* elements (*p* > *q*). The observed signals can be modeled as a superposition of source signals corrupted by additive circular Gaussian noise, which can be written as:

(1) x=As+n

where ***A*** is the *p* × *q* array steering matrix composed of *q* linearly independent column vectors, ***s*** is the *q*-dimensional source signal vector with nonsingular covariance matrix ***R**_S_* = *E* [***ss****^H^*] where (.)*^H^* stands for conjugate transpose, ***n*** is the source-independent noise vector with zero mean and covariance σ*^2^**I*** where ***I*** is the *p* × *p* identity matrix. Signals and noises are assumed to be i.i.d. and complex circular Gaussian distributed. The *p* × *p* population covariance matrix ***R*** is calculated as:

(2) $$R=E[xx^{H}]=AR_{S}A^{H}+\sigma^{2}I$$

whose population eigenvalues in descending order are given by:

(3) $$\lambda_{1}\geq \lambda_{2}\geq ...\geq \lambda_{q}>\lambda_{q+1}=...=\lambda_{p}=\sigma^{2}$$

The first *q* eigenvalues of ***R*** are contributed by both the source signals and the noise, which are called the signal eigenvalues. The last *p* – *q* eigenvalues are contributed by noise only, which are called the noise eigenvalues. The population covariance matrix can be estimated using the sample covariance matrix $\hat{R}$:

(4) $$\hat{R}=\frac{1}{N}\sum_{i=1}^{N}x_{i}x_{i}^{H}$$

where ***x_1_***, ... , ***x_N_*** are the independent and identically distributed snapshots of ***x***. The corresponding sample eigenvalues of $\hat{R}$ in descending order are given by:

(5) $$l_{1}>l_{2}>...>l_{q}>l_{q+1}>...>l_{p}\;$$

### 2.2. Source Enumeration of MDL

Assuming that all the observations are i.i.d. complex circular Gaussian random vectors with zero mean, the MDL estimator formulation is given by:

(6) $$MDL(k)=-L(k)+y(k)=N(p-k)\ln\frac{A_{k}}{G_{k}}+\frac{1}{2}\left[k(2p-k)+1\right]\ln N$$

where *L*(*k*) is the log-likelihood term, *y*(*k*) is the penalty term:

(7) $$A_{k}≜\frac{1}{p-k}\sum_{i=k+1}^{p}l_{i}$$

and:

(8) $$G_{k}≜\prod_{i=k+1}^{p}l_{i}^{1/(p-k)}$$

represent the arithmetic and geometric means of the last *p* – *k* eigenvalues respectively. The estimated source number is denoted by $\hat{q}$ which can be derived as follows:

(9) $$\hat{q}=\underset{k}{\arg\min}MDL(k),k=0,...,p-1$$

Let *H_q_* denote the hypothesis that the true number of sources is *q*. The probability of incorrect estimation *P_e_* is defined as:

(10) $$P_{e}=P\left(\hat{q}\neq q|H_{q}\right)=P_{m}+P_{f}$$

where the probability of missed detection *P_m_* is defined as:

(11) $$P_{m}=P\left(\hat{q}<q|H_{q}\right)$$

and the probability of false alarm *P_f_* is defined as:

(12) $$P_{f}=P\left(\hat{q}>q|H_{q}\right)$$

Ding and Kay [35] have proven that MDL is inconsistent at high SNR with a finite sample size. However, for the relatively large penalty term, MDL has a trend of underestimation of the source number at low SNR. For example, in the setting of *p* = 5, *q* = 2, *N* = 50 and SNR = 3 dB, the probability of false alarm *P_f_* is 0.0013 while the probability of underestimation *P_m_* is 0.5029 based on a 10,000 trial Monte Carlo simulation. Zhang *et al.*, showed in [18] that for a moderate number of snapshots, the probability of false alarm using the MDL criterion is approaching zero. So the probability of incorrect estimation *P_e_* is dominated by the probability of underestimation *P_m_*, which can be expressed as:

(13) $$P_{e}\approx P_{m}=P(\hat{q}<q|H_{q})\approx P\left(MDL(q-1)<MDL(q)|H_{q}\right)$$

Using Equation (6) in Equation (13) we obtain:

(14) $$P_{m}\approx P\left(\frac{A_{q-1}^{p-q+1}}{l_{q}A_{q}^{p-q}}<\exp\left(\frac{(2p-2q+1)\ln N}{2N}\right)\right)$$

According to the definition of *A_k_* in Equation (7), we can rewrite *A_q−1_* as:

(15) $$A_{q-1}=\frac{\sum_{i=q}^{p}l_{i}}{p-q+1}=\frac{l_{q}+\sum_{i=q+1}^{p}l_{i}}{p-q+1}\text{=}\frac{l_{q}+(p-q)A_{q}}{p-q+1}$$

In order to simplify Equation (14), we define:

(16) $$\rho_{i}=l_{i}/A_{i},\rho_{i}>1$$

when *i* = *q*, we will have:

(17) $$\rho_{q}=l_{q}/A_{q},\rho_{q}>1$$

Substituting Equations (15) and (17) into Equation (14), we can get:

(18) $$P_{m}\approx P\left(f_{1}(\rho_{q})<C_{1}\right)$$

where:

(19) $$wf_{1}(x)≜\left(x+p-q\right)x^{-\frac{1}{p-q+1}}$$

and:

(20) $$C_{1}=(p-q+1)\exp\left(\frac{(2p-2q+1)\ln N}{2N(p-q+1)}\right)$$

The function *f_1_*(*x*) is a monotonically increasing function in the region of *x* > 1, therefore we can transform Equation (17) into a simpler form as:

(21) $$P_{m}\approx P\left(\rho_{q}<\rho_{C1}\right)$$

in which:

(22) $$f_{1}(\rho_{C1})=C_{1}$$

Since Equation (21) cannot be solved analytically, we can use the Newton-Raphson method to find a very accurate solution of $\rho_{C1}$ numerically from the initial value derived by binomial expansion:

(23) $$\rho_{C1}^{0}=D_{1}+\sqrt{D_{1}^{2}-1}$$

where:

(24) $$D_{1}={\left(\frac{C_{1}}{p-q+1}\right)}^{p-q+1}$$

For a more complicated situation, we will discuss the cases of multiple-missed detection as follows. Consider that the true source number *q* is underestimated by a number greater than or equal to *d*, we define the corresponding probability as:

(25) $$P_{md}=P\left(\hat{q}\leq q-d|H_{q}\right),d<q$$

in order to distinguish the probabilities for different underestimated source numbers. So:

(26) $$P_{md}\approx P\left(MDL(q-d)<MDL(q-d+1)|H_{q}\right)$$

By using Equation (6) in Equation (26), we can derive:

(27) $$P_{md}\approx P\left(\frac{A_{q-d}^{p-q+d}}{l_{q-d+1}A_{q-d+1}^{p-q+d-1}}<\exp\left(\frac{(2p-2q+2d-1)\ln N}{2N}\right)\right)$$

Let *i* = *q* – *d* + 1 in Equation (16), we will have:

(28) $$\rho_{q-d+1}=l_{q-d+1}/A_{q-d+1},\rho_{q-d+1}>1$$

and obtain a simpler form of Equation (26):

(29) $$P_{md}\approx P\left(f_{d}(\rho_{q-d+1})<C_{d}\right)$$

where:

(30) $$f_{d}(x)≜\left(x+p-q+d-1\right)x^{-\frac{1}{p-q+d}}$$

(31) $$C_{d}=(p-q+d)\exp\left(\frac{(2p-2q+2d-1)\ln N}{2N(p-q+d)}\right)$$

Since *f_d_*(*x*) is also a monotonically increasing function in the region of *x* > 1, Equation (29) can be rewritten as:

(32) $$P_{md}\approx P\left(\rho_{q-d+1}<\rho_{Cd}\right)$$

in which:

(33) $$f_{1}(\rho_{Cd})=C_{d}$$

The threshold $\rho_{Cd}$ can be calculated numerically from the initial value:

(34) $$\rho_{Cd}^{0}=D_{d}+\sqrt{D_{d}^{2}-1}$$

where:

(35) $$D_{d}={\left(\frac{C_{d}}{p-q+d}\right)}^{p-q+d}$$

We can find that *P_m_* is a particular form of *P_md_* when *d* = 1. So the problem of underestimation probability turns into the statistical performance analysis of ρ*_i_* which will be discussed in the next section. The expectation of the estimated source number can be calculated by:

(36) $$E(\hat{q})=q(1-P_{m1})+P_{m(q-1)}+\sum_{d=1}^{q-2}\left(q-d)P_{md}(1-P_{m(d+1)}\right),\hat{q}\in [1,q]$$

which would be an effective indicator of the extent of underestimation.

## 3. Performance Analysis of Multiple-Missed Detection

According to Equation (16), the statistics of ρ*_i_* are determined by the distributions of *l_i_* and *A_i_*. Many researches have been done on the statistical properties of the signal sample eigenvalue *l_i_* and the arithmetic mean of the noise sample eigenvalues *A_q_*, and can be mainly divided into multivariate statistical theory [28], random matrix theory [29] and Lawley’s theory [34]. The multivariate statistical theory is derived based on large sample asymptotics and requires a large value of sample size *N*. Random matrix theory has been proposed to investigate the spectral properties of random matrices with the assumption of high-dimension and large sample asymptotic regime. Nadakuditi and Edelman have concluded in [32] that for a signal-free sample covariance matrix formed from a *p* × *N* matrix of observations with i.i.d. Gaussian samples of zero mean and identical variance $\sigma^{2}$, the sample eigenvalues will follow the Marchenko-Pastur distribution and their arithmetic mean will converge to Gaussian distribution asymptotically as *p*, *N* →∞ with p/N→c∈(0,∞), *i.e.*,

(37) $$p\left(\frac{1}{p}\sum_{i=1}^{p}l_{i}-\sigma^{2}\right)\overset{D}{\to }N\left(0,\frac{2\sigma^{4}c}{\beta }\right)$$

where $\overset{D}{\to }$ denotes convergence in distribution and β = 1 or 2 for real or complex values respectively. For a *q*-signal-bearing case defined in Section 2.1, as *p*, *N* → ∞ with p/N→c∈(0,∞), if all the signal eigenvalues of the population covariance matrix are larger than the critical value $\sigma^{2}(1+\sqrt{c})$ where $\sigma^{2}$ stands for the value of noise eigenvalue, which means no phase transition phenomenon, the distribution of signal sample eigenvalues can be described as following. If the signal eigenvalue $\lambda_{i}>\sigma^{2}(1+\sqrt{c})$ has multiplicity of one for *i* ≤ *q* and $\sqrt{N}\left|c-p/N\right|\to 0$, the distribution of *l_i_* converges to Gaussian distribution as:

(38) $$\sqrt{N}\left(l_{i}-\lambda_{i}\left(1+\frac{\sigma^{2}c}{\lambda_{i}-\sigma^{2}}\right)\right)\overset{D}{\to }N\left(0,\frac{2}{\beta }\lambda_{i}^{2}\left(1-\frac{c\sigma^{4}}{{\left(\lambda_{i}-\sigma^{2}\right)}^{2}}\right)\right)$$

The distribution analysis of *A_q_* can be performed as the arithmetic mean of the sample eigenvalues of a signal-free sample covariance matrix formed from a (*p* – *q*) × *N* matrix of observations. Although the random matrix theory is derived under the assumption of large dimension, some simulation results have shown that it may also work well in some low-dimension cases. Lawley has constructed a matrix with the same eigenvalues as the sample covariance matrix by using the sampling errors. By comparing the diagonal elements, the statistics of sample eigenvalues are derived under the assumption of Gaussian distribution. All the three theories assume that *l_i_* and *A_q_* follow Gaussian distribution asymptotically and the expectations and variances are listed in Table 1 for comparison.

Note that the expectations and variances of *l_i_* in random matrix theory and Lawley’s theory have an additional term compared with those in multivariate statistical theory. The random matrix theory has taken the disturbance from noise subspace into account while Lawley’s theory has considered the interactions between signal eigenvalues in addition. When the sample size *N* is sufficiently large, the additional terms will diminish to zero and the distributions of all the three theories will equal to each other. Similarly, the expectation of *A_q_* in Lawley’s theory is different from the others for including the bias induced by the signal eigenvalues. Thus, we employ the expectation and variance of *l_i_* in Lawley’s theory for an accurate analysis, and additional terms with the order higher than *O*(*N*^−1^) in the expectations or *O*(*N*^−2^) for variances are omitted as they are relatively small and decay rapidly with increasing sample size. The phenomenon of phase transition is not considered for simplicity in this paper.

**Table 1:** Comparison of three theories on the expectations and variances of *l_i_* and *A_q_*.

	Multivariate Statistics	Random Matrix	Lawley
*E*(*l_i_*) *^1^	$\lambda_{i}$	$\lambda_{i}+\frac{c\lambda_{i}\sigma^{2}}{\lambda_{i}-\sigma^{2}}$ *^3^	$\lambda_{i}+\sum_{j\neq i}^{p}\frac{\lambda_{i}\lambda_{j}}{N(\lambda_{i}-\lambda_{j})}$
*Var*(*l_i_*) *^1^	$\frac{\lambda_{i}^{2}}{N}$	$\frac{2\lambda_{i}^{2}}{\beta N}\left[1-\frac{c\sigma^{4}}{{\left(\lambda_{i}-\sigma^{2}\right)}^{2}}\right]$ *^2,^*^3^	$\frac{2\lambda_{i}^{2}}{\beta N}\left[1-\frac{1}{N}\sum_{j\neq i}^{p}{\left(\frac{\lambda_{j}}{\lambda_{i}-\lambda_{j}}\right)}^{2}\right]$ *^2^
*E*(*A_q_*)	$\sigma^{2}$	$\sigma^{2}$	$\sigma^{2}-\sum_{i=1}^{q}\frac{\lambda_{i}\sigma^{2}}{N(\lambda_{i}-\sigma^{2})}$
*Var*(*A_q_*)	$\frac{\sigma^{4}}{N(p-q)}$	$\frac{2\sigma^{4}}{\beta N(p-q)}$ *^2^	/

*^1^ *i ≤ q* for the expectations and variances of *l_i_. E*(.) and *Var*(.) are the mathematical expectation and variance respectively; *^2^ β = 1 for real-valued signals and β = 2 for complex-valued signals. The original formula of Lawley’s *Var*(*l_i_*) without β is revised here; *^3^ *c* is a positive finite value when *p*, *N* →∞, *p*/*N* → *c.*

The expectation and variance of *A_i_* are given only under the condition of *i* = *q* in above theories. Now we will discuss the distributive property of *A_i_* to get general expressions when *i* < *q*. According to the definition of *A_i_* in Equation (7), we can rewrite *A_i_* as:

(39) $$A_{i}=\frac{1}{p-i}\left((p-q)A_{q}+\sum_{j=i+1}^{q}l_{j}\right)$$

Noting that:

(40) $$E\left((p-i)A_{i}+\sum_{j=1}^{i}l_{i}\right)=E(tr(\hat{R}))=tr(R)=\sum_{j=1}^{p}l_{j}$$

we can obtain:

(41) $$E(A_{i})=\frac{1}{p-i}\left\{\sum_{j=i+1}^{q}\left[\lambda_{j}+\sum_{k=1}^{i}\frac{\lambda_{j}\lambda_{k}}{N(\lambda_{j}-\lambda_{k})}\right]-\sum_{j=1}^{i}\frac{\lambda_{j}\sigma^{2}(p-q)}{N(\lambda_{j}-\sigma^{2})}+(p-q)\sigma^{2}\right\},i\leq q$$

which equals to the expectation of *A_q_* in Lawley’s theory as *i* = *q*. When *i* < *q*, *A_i_* will contain signal sample eigenvalues. We assume that the covariance between signal and noise sample eigenvalues can be ignored. The covariance between signal sample eigenvalues is given in [34] as:

(42) $$Cov(l_{i},l_{j})=\frac{2\lambda_{i}^{2}\lambda_{j}^{2}}{\beta N^{2}{\left(\lambda_{i}-\lambda_{j}\right)}^{2}},i\neq j，i,j\leq q$$

According to the properties of variance and complex circular Gaussian assumption, *A_i_* is also asymptotically Gaussian distributed and the variance can be derived as:

(43) $$Var(A_{i})=Var\left(\frac{p-q}{p-i}A_{q}+\frac{\sum_{j=i+1}^{q}l_{j}}{p-i}\right)=\frac{{\left(p-q\right)}^{2}Var(A_{q})}{{\left(p-i\right)}^{2}}+\sum_{j=i+1}^{q}\frac{Var(l_{j})}{{\left(p-i\right)}^{2}}+2\sum_{j=i+1}^{q}\sum_{j<k\leq q}\frac{Cov(l_{j},l_{k})}{{\left(p-i\right)}^{2}}$$

Note that Equation (43) equals to the variance of *A_q_* when *i* = *q*, so Equation (43) is the general expression of the variance of *A_i_* for *i* ≤ *q*. Since the distributions of *l_i_* and *A_i_* are asymptotically Gaussian, the distribution of ρ*_i_* defined in Equation (16) would follow a ratio distribution of two Gaussian random variables. The probability density function of the ratio of two correlated Gaussian random variables has been derived by Hinkley in [36]. The correlation coefficient *r_i_* between *l_i_* and *A_i_* can be derived as:

(44) $$r_{i}=\frac{Cov(l_{i},A_{i})}{\sqrt{Var(l_{i})Var(A_{i})}}=\frac{1}{(p-i)\sqrt{Var(l_{i})Var(A_{i})}}\sum_{j=i+1}^{q}Cov(l_{i},l_{j})，i<q$$

So the probability density function *f*(ρ*_i_*) of ρ*_i_* can be obtained by using Equation (1) of [36]. Furthermore, the probability of multiple-missed detection *P_md_* can be calculated as:

(45) $$P_{md}=F(\rho_{Cd})=\int_{-\infty }^{\rho_{Cd}}f(\rho_{i})d\rho_{i}$$

where:

(46) $$F(x)=\int_{-\infty }^{x}f(t)dt$$

is the cumulative distribution function of the Gaussian ratio distribution.

## 4. Simulation Setup and Numerical Results

In the numerical simulations, a uniform linear array with an inter-sensor spacing of half-wavelength is employed. The observed signals are assumed to be uncorrelated complex circular Gaussian source signals contaminated by additive complex circular white Gaussian noise and some results may be invalid for arbitrary complex signals. The numbers of samples, sensors, true sources and underestimated sources are denoted by *N*, *p*, *q* and *d* respectively. The DOAs are denoted by the vector **θ** and SNR is short for the signal-to-noise ratio. The probability of an underestimated source number greater than or equal to *d* is denoted by *P_md_* and the expectation of the estimated source number is denoted by $E(\hat{q})$. All the simulation results are obtained based on 10,000 Monte Carlo trials.

### 4.1. Evaluation of the Proposed Method for Underestimation Analysis

The methods presented in [25,26,27] are used for comparison which based on the statistical analysis of sample eigenvalues as well. Haddadi *et al.* [25] use the expectations in Lawley’s theory and the variance in multivariate statistical theory with the neglect of the variances of noise sample eigenvalues while Huang *et al.* [26] employ the variance in random matrix theory. Lu and Zoubir [27] have incorporated the expectations in Lawley’s theory and the variances in random matrix theory to predict the estimation results of MDL precisely. All the four procedures use the same expectations of *l_i_* and *A_q_* whereas different variances are selected. A comparison among different procedures is shown in Table 2.

**Figure 1:**
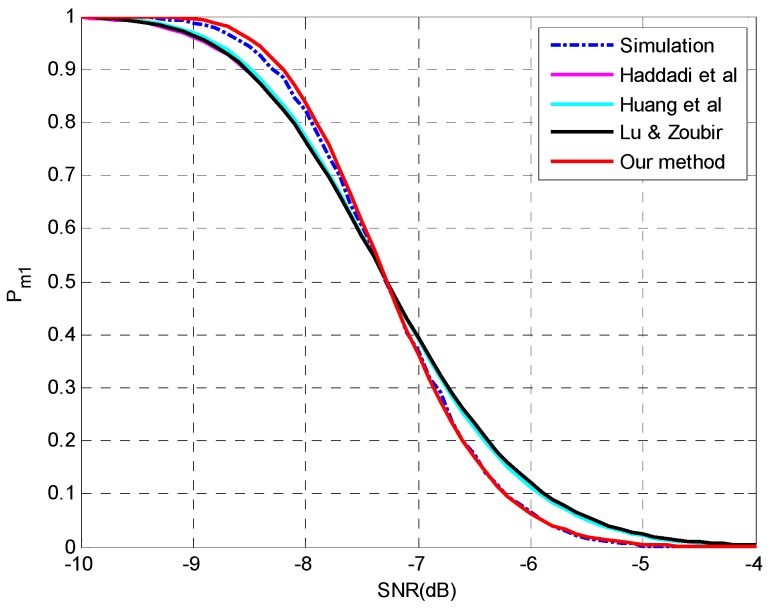
The probability of underestimation *P_m1_ versus* SNR with *N* = 50, *p* = 20, *q* = 3, **θ** = {−5°, 0°, 5°}.

**Figure 2:**
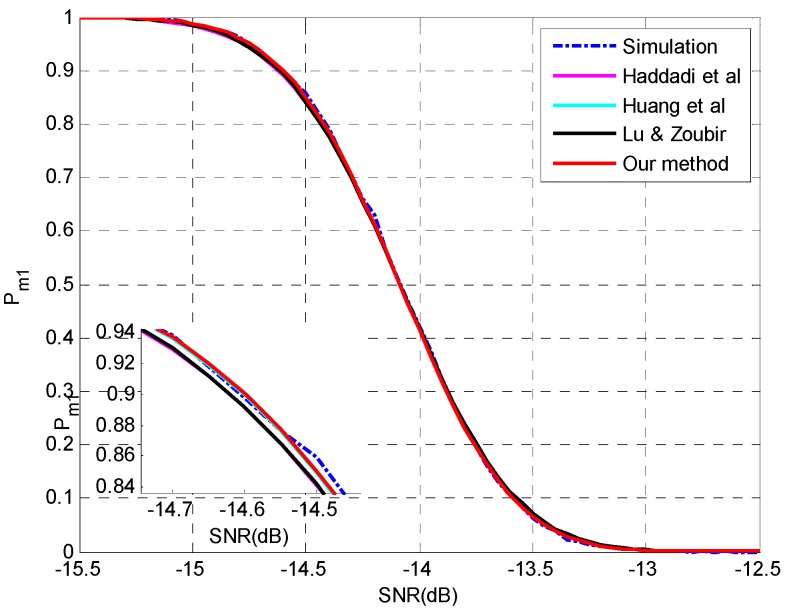
The probability of underestimation *P_m1_ versus* SNR and details with *N* = 1000, *p* = 20, *q* = 3, **θ** = {−5°, 0°, 5°}.

**Figure 3:**
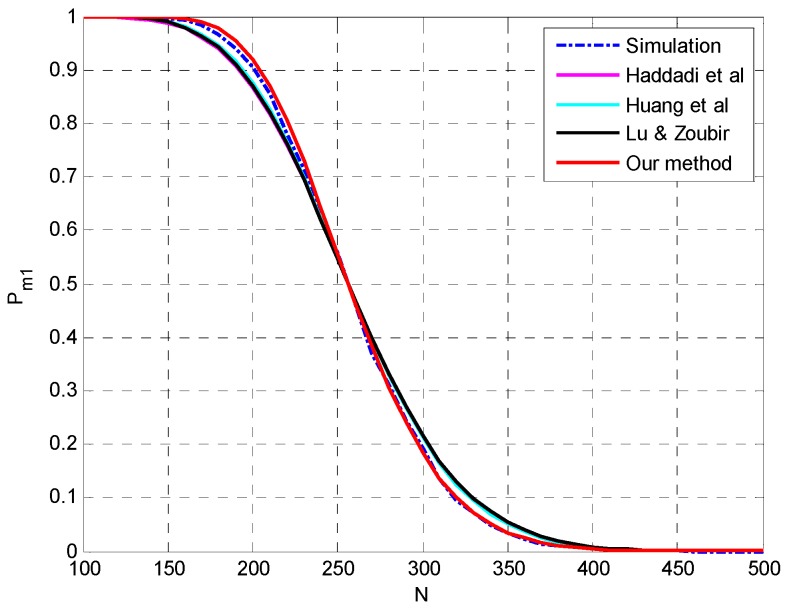
The probability of underestimation *P_m1_ versus N* with *p* = 30, *q* = 3, **θ** = {−5°, 5°, 10°}, SNR = −12 dB.

**Figure 4:**
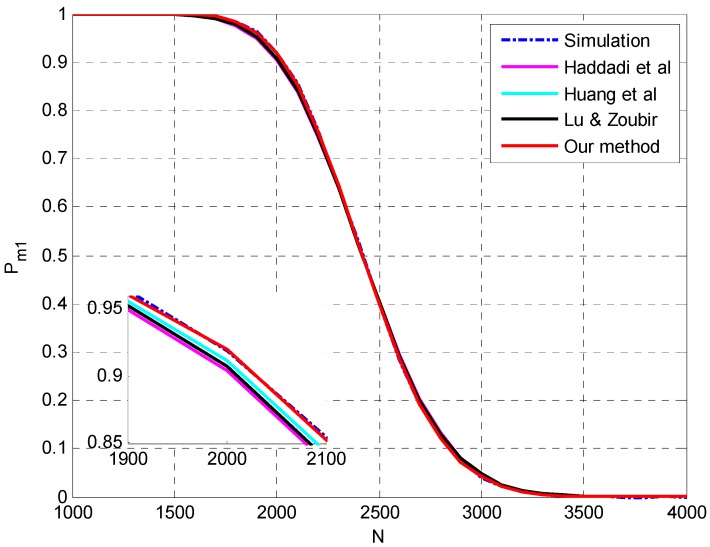
The probability of underestimation *P_m1_ versus N* and details with *p* = 30, *q* = 3, **θ** = {−5°, 5°, 10°}, SNR = −17 dB.

Four experimental settings are listed as following by varying the SNR or the sample size *N*:

**Setting 1** (see Figure 1): *N* = 50, *p* = 20, *q* = 3, **θ** = {−5°, 0°, 5°}, SNR = [−10,−4] dB.
**Setting 2** (see Figure 2): *N* = 1000, *p* = 20, *q* = 3, **θ** = {−5°, 0°, 5°}, SNR = [−15.5,−12.5] dB.
**Setting 3** (see Figure 3): *N* = [100,500], *p* = 30, *q* = 3, **θ** = {−5°, 5°, 10°}, SNR = −12 dB.
**Setting 4** (see Figure 4): *N* = [1000,4000], *p* = 30, *q* = 3, **θ** = {−5°, 5°, 10°}, SNR = −17 dB.

**Table 2:** Theoretical comparison of the four procedures.

	Haddadi *et al.*	Huang *et al.*	Lu &Zoubir	Ours
*E*(*l_q_*)	Lawley	Lawley	Lawley	Lawley
*Var*(*l_q_*)	Multivariate Statistics	Random Matrix	Random Matrix	Lawley
*E*(*A_q_*)	Lawley	Lawley	Lawley	Lawley
*Var*(*A_q_*)	- *	-	Random Matrix	Random Matrix

* Means this term has been neglected.

As shown in Figure 1, the proposed method shows the best agreement with simulation results and outperforms the others. It is worth noting that the curves nearly intersect at the same point of *P_m_* = 0.5 with different shapes which may correspond to the expectations and variances, respectively. The methods in [25,27] have very similar performance since the former uses a larger variance of signal sample eigenvalue while the latter considered the variance of noise sample eigenvalues as a counteract. An inconspicuous difference is found for the method in [26] due to the consideration of only the interactions between signal and noise subspaces.

The simulation results at a large sample size are presented in Figure 2. All the four methods match the simulation results pretty well and the superiority of the proposed method and the method in [26] can be confirmed in the details of Figure 2. The accurate prediction by the method in [26] may be attributed to the reason that the ignorance of the variance of noise sample eigenvalues may compensate the interactions between signal eigenvalues. Similar results are presented in Figure 3 and Figure 4 by varying the sample size *N* instead of SNR and the proposed method outperforms the other methods.

The outstanding performance of the proposed method can be attributed to the fact that the interactions between signal eigenvalues have been taken into consideration in estimating the variance of signal sample eigenvalue. The performance of the methods in [25,26,27] is dependent of the sample size in the simulation settings, since they ignore the interactions between signal eigenvalues which are sensitive to the sample size. In the cases when the sample size is sufficiently large, all the methods are capable to yield satisfactory results. However, MDL is widely used in practical applications where only a limited number of samples is available. Thus, the proposed method is of more practical value for its accuracy in such cases.

### 4.2. Evaluation of the Analysis on Multiple-Missed Detection

**Figure 5:**
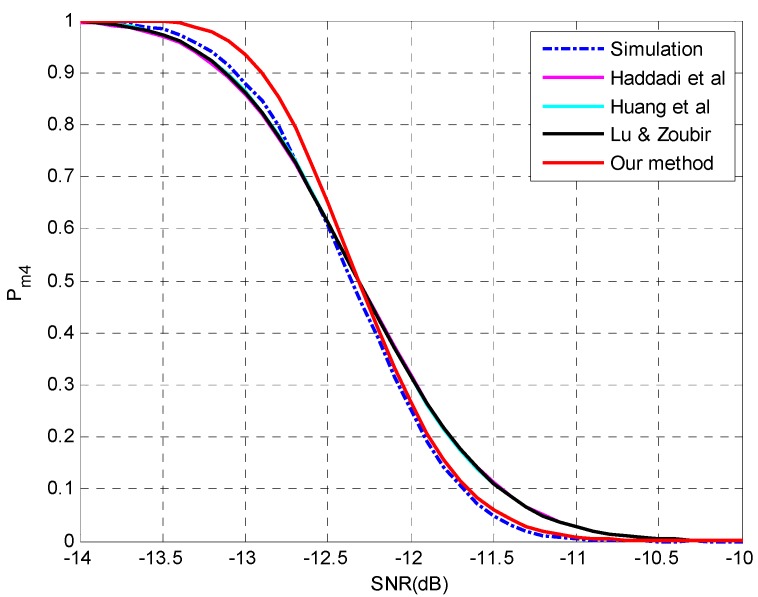
The probability of underestimation *P_m4_ versus* SNR with *N* = 150, *p* = 30, *q* = 5, **θ** = {−7°, −5°, 0°, 3°, 11°}, *d* = 4.

**Figure 6:**
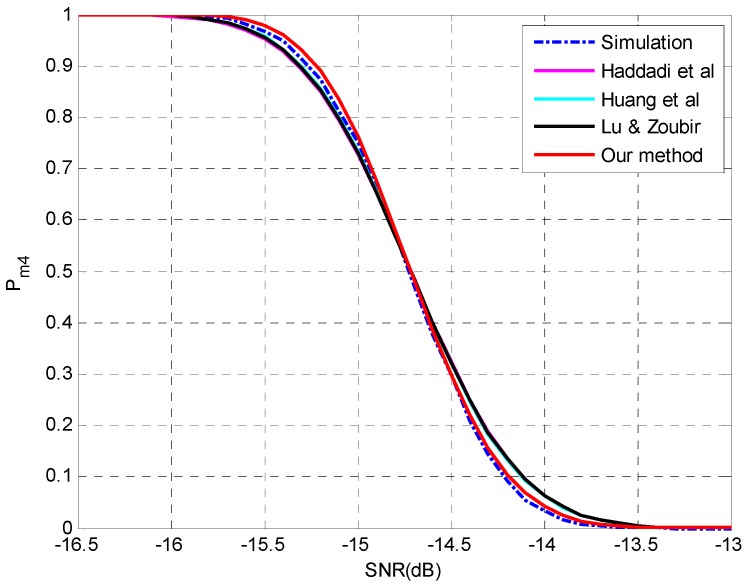
The probability of underestimation *P_m4_ versus* SNR with *N* = 400, *p* = 30, *q* = 5, **θ** = {−7°, −5°, 0°, 3°, 11°}, *d* = 4.

To evaluate the performance of the proposed method for multiple-missed detection, the algorithms in [25,26,27] have been adapted by the proposed approach as reference methods. Experimental settings are listed as following:

**Setting 5** (see Figure 5): *N* = 150, *p* = 30, *q* = 5, **θ** = {−7°, −5°, 0°, 3°, 11°}, SNR = [−14,−10] dB, *d* = 4.
**Setting 6** (see Figure 6): *N* = 400, *p* = 30, *q* = 5, **θ** = {−7°, −5°, 0°, 3°, 11°}, SNR = [−16.5,−13] dB, *d* = 4.

The simulation results are shown in Figure 5 and Figure 6 at different sample sizes. As indicated in Figure 5, the proposed method is more accurate in the region of *P_m_* < 0.5 while the other three methods perform better in the region of *P_m_* > 0.5. When the sample size is increased to 400, the discrepancy among the methods become smaller and the superiority of the proposed method is reconfirmed in the entire region. We attribute this phenomenon to the omission of the higher-order terms, since a bias can be found between the simulation results and all the predictions in Figure 5, while the bias is perfectly rectified at a slightly larger sample size in Figure 6. The accumulated error by the neglect of higher-orders terms would affect the performance analysis for multiple-missed detection and a moderate sample size may be required for an accurate estimation.

**Figure 7:**
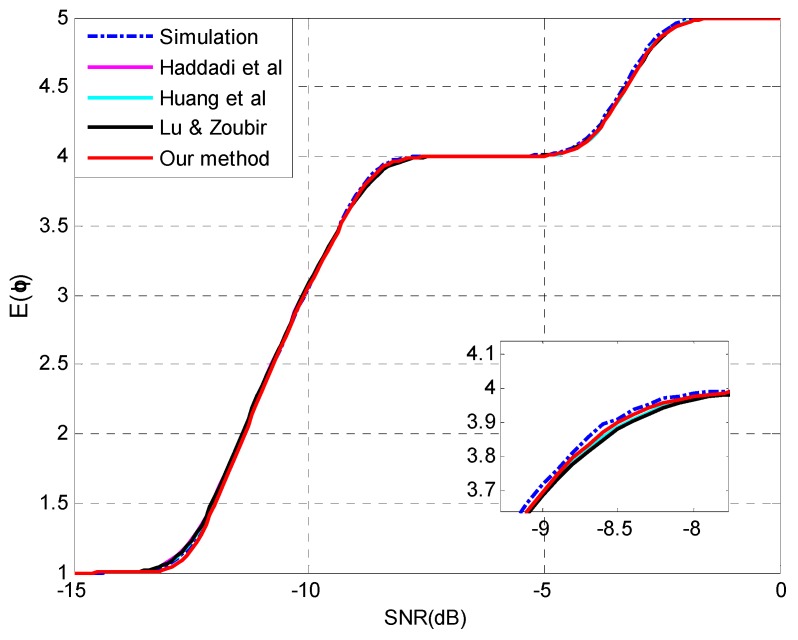
The expectation of the estimated source number $E(\hat{q})$ *versus* SNR and details with *N* = 150, *p* = 20, *q* = 5, **θ** = {−10°, −6°, 0°, 6°, 8°}.

**Figure 8:**
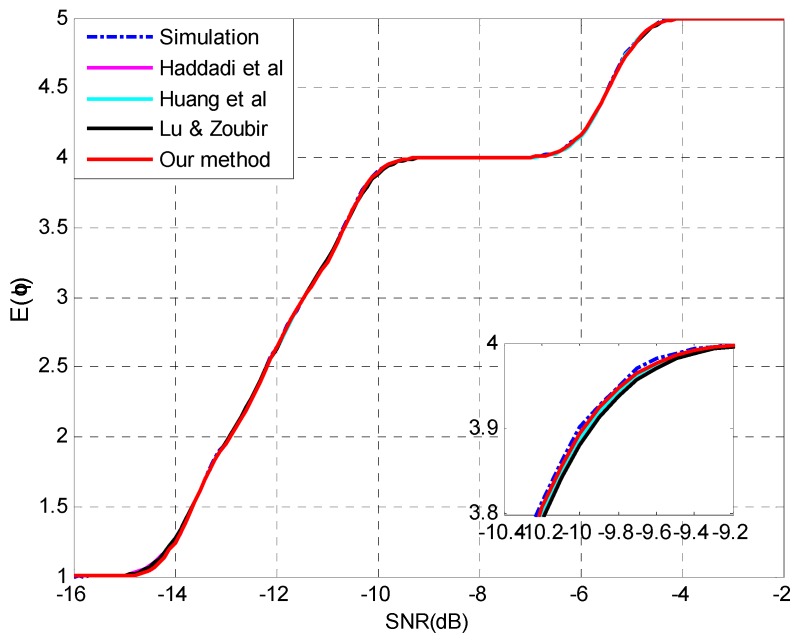
The expectation of the estimated source number $E(\hat{q})$ *versus* SNR and details with *N* = 300, *p* = 20, *q* = 5, **θ** = {−11°, −7°, 0°, 2°, 10°}.

To assess the performance deterioration of enumeration with the degradation of SNR, we use the expectation of the estimated source number as the indicator which is calculated by Equation (36). Experimental settings are listed as follows:

**Setting 7** (see Figure 7): *N* = 150, *p* = 20, *q* = 5, **θ** = {−10°, −6°, 0°, 6°, 8°}, SNR = [−15,0] dB.
**Setting 8** (see Figure 8): *N* = 300, *p* = 20, *q* = 5, **θ** = {−11°, −7°, 0°, 2°, 10°}, SNR = [−16,−2] dB.

Figure 7 and Figure 8 show that all the four methods are able to perform the analysis for multiple-missed detection precisely and the capability of the proposed approach for multiple-missed detection is verified. The mean absolute error (MAE) between the prediction and simulation results is selected to assess the performance of different methods quantitatively. The MAEs are listed in Table 3 and the superiority of our method can be confirmed.

**Table 3:** MAE of the methods in predicting the enumeration results.

MAE	Haddadi *et al*.	Huang *et al*.	Lu & Zoubir	Our Method
Setting 7	0.0143	0.0123	0.0140	0.0110
Setting 8	0.0054	0.0047	0.0055	0.0043

## 5. Conclusions

This paper presents an accurate performance analysis for the underestimation performance of the MDL source enumeration method at a finite sample size in array processing. Theoretical derivations and statistical analyses have been performed with the consideration of the interactions between signal eigenvalues to obtain an improved estimation of the probability of underestimation. A new approach is also proposed to evaluate the performance of multiple-missed detection cases by ratio distribution analysis and can be employed by the eigenvalue-analyzing methods. Simulation results show the superiority of the proposed analysis, and verify the ability of the proposed approach in evaluating the deterioration of enumeration performance with the degradation of SNR, which may be a valuable reference for practical applications.
